# DECIPHERING RACIAL AND ETHNIC DISPARITIES IN DEMENTIA AND COGNITIVE FUNCTION: A POLYSOCIAL SCORE APPROACH

**DOI:** 10.1093/geroni/igac059.001

**Published:** 2022-12-20

**Authors:** Chenkai Wu, Yongjing Ping, Xi Chen, Michelle Odden, Matthew Prina

**Affiliations:** Duke Kunshan University, Kunshan, Jiangsu, China (People's Republic); Duke-NUS, Singapore, Singapore; Yale University, New Haven, Connecticut, United States; Stanford University, Stanford, California, United States; King's College London, London, England, United Kingdom

## Abstract

The burden of dementia has been increasing rapidly in the US and huge racial/ethnic disparities exist. We examined whether social environment, measured in a comprehensive way (polysocial score approach), could modify the racial and ethnic differences in dementia. Data are from the Health and Retirement Study; 12,836 adults aged at least 65 years were included in the analysis. We assessed cognitive function by a modified version of the telephone interview for cognitive status. We included 24 social factors from five categories (economic stability, neighborhood environment, education, community/social context, and healthcare system) and used forward stepwise regression to screen for important ones. Polysocial score was created using 11 social factors and was classified as low (< 25), intermediate (25-31), and high (32+). We used the multivariable Poisson regression to estimate the incidence rate of dementia by three polysocial score categories and evaluate the interaction between race/ethnicity (non-Hispanic white, non-Hispanic Black, and Hispanic) and the polysocial score. Higher polysocial score is associated with a lower dementia rate in the overall sample and each racial/ethnic group. We found an additive interaction between race/ethnicity and polysocial score categories. In the low polysocial score group, Whites and Hispanics had substantially higher dementia rates than non-Hispanic Blacks (difference was 20.3 and 15.8 per 1,000 persons-years, respectively). These differences significantly attenuated and were no longer significant in the high polysocial score group (4.9 and 5.5 per 1,000 persons-years, respectively). The polysocial score approach offers a new opportunity to explain the racial/ethnic disparities in cognitive function among older adults.

